# Landscape of CDKN1B Mutations in Luminal Breast Cancer and Other Hormone-Driven Human Tumors

**DOI:** 10.3389/fendo.2018.00393

**Published:** 2018-07-17

**Authors:** Martina Cusan, Giorgia Mungo, Mara De Marco Zompit, Ilenia Segatto, Barbara Belletti, Gustavo Baldassarre

**Affiliations:** Division of Molecular Oncology, CRO of Aviano, IRCCS, National Cancer Institute, Aviano, Italy

**Keywords:** p27^Kip1^ protein, *CDKN1B* gene, somatic mutation, germinal mutation, driver mutation, passenger mutation, Intrinsically disordered protein/region

## Abstract

The *CDKN1B* gene encodes for the p27^Kip1^ protein, firstly characterized as a cyclin dependent kinase (CDK)-inhibitor. Germline *CDKN1B* pathogenic variants have been described in hereditary tumors, such as multiple endocrine neoplasia (MEN)-like syndromes and familial prostate cancer. Despite its central role in tumor progression, for a long time it has been proposed that *CDKN1B* was very rarely somatically mutated in human cancer and that its expression levels were almost exclusively regulated at post-transcriptional level. Yet, the advent of massive parallel sequencing has partially subverted this general understanding demonstrating that, at least in some types of cancer, *CDKN1B* is mutated in a significant percentage of analyzed samples. Recent works have demonstrated that *CDKN1B* can be genetically inactivated and this occurs particularly in sporadic luminal breast cancer, prostate cancer and small intestine neuroendocrine tumors. However, a clear picture of the extent and significance of *CDKN1B* mutations in human malignances is still lacking. To fill this gap, we interrogated the COSMIC, ICGC, cBioPortal, and TRANSFAC data portals and current literature in PubMed, and reviewed the mutational spectrum of *CDKN1B* in human cancers, interpreting the possible impact of these mutations on p27^Kip1^ protein function and tumor onset and progression.

## Introduction

**Figure 1:**
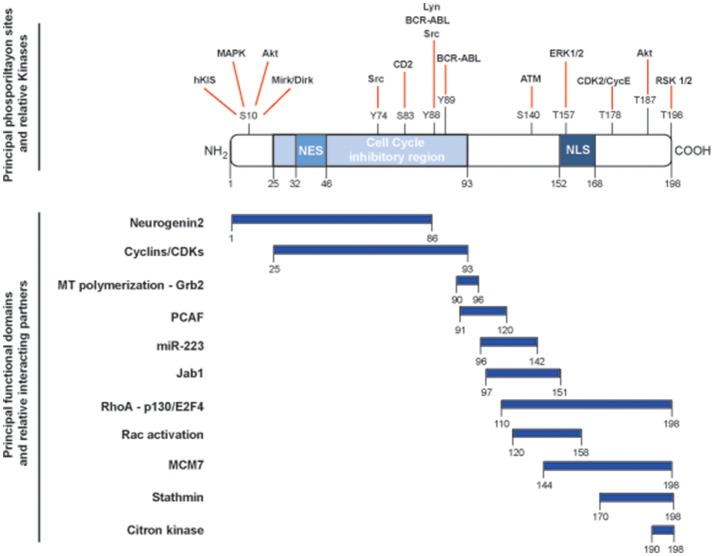
Schematic representation of the main functional domains and phosphorylation sites of p27. p27 protein is composed by 198 amino acids and contains a nuclear exportation signal (NES) at the N-terminus and a nuclear localization signal (NLS) at the C-terminus. Key phosphorylation sites and corresponding kinases are depicted in the upper part of the figure and linked with red lines. The cell cycle inhibitory region is comprised between amino acids 25and 93 and is necessary for the binding to cyclin/CDK complexes. Known functional domains and relative interacting protein/microRNA are reported below and highlighted by blue rectangles.

The *CDKN1B* gene encodes for the p27^Kip1^ protein (hereafter p27), firstly characterized as an inhibitor of cell cycle progression for its ability to bind and regulate a broad range of cyclin-CDK (cyclin-dependent kinase) complexes (1). *CDKN1B* belongs to a family of CDK inhibitor (CKI) genes that also comprises *CDKN1A* (encoding for p21^Waf1^) and *CDKN1C* (encoding for p57^Kip2^). The three CKI proteins share a region of high homology at their N-terminal portion, encompassing the cyclin- and the CDK-binding domains (2, 3). This region confers the ability to bind and inhibit, although with different stoichiometry, all cyclin/CDKs complexes, eventually controlling progression through the cell cycle. Each member of the family has specific functions and, in this context, p27 has been more prominently indicated as a sensor of external stimuli in driving the decision of the cell to enter or not the cell cycle and, eventually, divide (4). However, depicting p27 only as a cell cycle regulating protein is an old representation of its cellular functions. Many studies have clearly demonstrated that p27 has the ability to interact with many different proteins and represents a target for many signal transduction pathways, thereby accomplishing a number of previously unexpected and so-called non-canonical functions (5) (Figure 1).

A central role in the determination of p27 interactions and functions is played by its subcellular localization. p27 shuttles from the nucleus to the cytoplasm, and *vice versa*, depending from the phase of the cell cycle and from the activation of specific signaling. Different functions can be played by p27 when localized in different subcellular compartment, including regulation of transcription, cytoskeleton organization, autophagy, cytokinesis, eventually impacting on cell proliferation, survival, differentiation, motility, and invasion (5).

These different activities of p27 are highly interconnected and it has been proven difficult to separate these functions only on the basis of specific interactions and/or subcellular localization, although this aspect has been a matter of intense study in the last decade. For instance, we reported that the cytoplasmic interaction of p27 C-terminal domain with the microtubule-destabilizing protein stathmin interferes with microtubule dynamics and regulates cellular migration (6). More recently, the study of p27 and stathmin double knock-out mice unveiled that the cytoplasmic interaction of p27 and stathmin also regulates cell cycle progression in a CDK-independent manner, acting on the H-Ras/MAPK pathway (7–9). Similarly, nuclear localization of p27 has been reported to be necessary for its tumor suppressive function in mice and it has been linked to the control of cell cycle progression *via* the binding with cyclins/CDK complexes (10). However, nuclear p27 localization is also necessary for embryonic stem cell differentiation and cell reprogramming into induced pluripotent stem cells (iPSCs), *via* the transcriptional repression of the SOX2 gene (11).

The fact that p27 plays many different functions and binds many different partners has stimulated the hypothesis that it could act either as oncogene or tumor suppressor gene, in a context-dependent or interaction-dependent manner (12). However this hypothesis has never been recapitulated in human tumors yet. Also the possibility that specific post-translational modifications, such as phosphorylation, ubiquitination or SUMOylation, could directly impact on p27 functions and/or on its interactions with other proteins is a scientific issue that might merit more investigations.

In this context, it is interesting to note that p27 is an intrinsically disordered protein (IDP) that adopts an extended conformation upon binding to cyclin A/CDK2 complex at the N-terminus while the C-terminus retains the characteristics of intrinsically disordered region (IDR) (13, 14). The presence of IDRs in a protein greatly weakens the old postulate that protein sequence determines protein structure and protein structure determines protein function (13). Indeed, in an interesting review of the literature, Babu proposed that IDRs increase the functional versatility of proteins and may contribute to human disease (13). The presence of IDRs in a protein allows for the promiscuous interaction with a large number of “partners” and/or facilitates protein function regulation *via* diverse upstream pathways and post-translational modifications.

Thanks to their conformational flexibility, IDPs are therefore excellent substrates to encode and decode information *via* post-translational modifications (13). This hypothesis is in line with the current knowledge on p27 protein functions and interactions (Figure 1) and supports the possibility that p27 is very well suited to perform signaling and regulatory functions in multiple contexts.

The *CDKN1B* gene was the first gene described as haplo-insufficient for tumor suppression, meaning that it did not follow the Knudson's “two-hit” theory. Animals lacking only one copy of *Cdkn1b* already displayed a tumor-prone phenotype, with increased tumor frequency and decreased latency when challenged with different carcinogens and spontaneously developed pituitary tumors with a mild penetrance, late in life (15). The complete ablation of *Cdkn1b* led to 20–30% increased body size respect to wild-type littermates, and predisposed to spontaneous pituitary adenomas and multiple organ hyperplasia (15, 16). Importantly, the phenotype observed in mice is fairly well recapitulated also in humans affected by the syndrome of multiple endocrine neoplasia (MEN) that carry germline *CDKN1B* mutations (17).

These molecular and preclinical evidences have acquired particular clinical interest in the last few years, when mutations of *CDKN1B* gene were unexpectedly identified as driver mutations in selected types of human cancers. Deletion, methylation or mutations of *CDKN1B* gene have been considered very rare for a long period of time. Consistently, loss of p27 in tumors has been always ascribed to an accelerated proteolysis (18, 19). However, the advent of massive parallel sequencing that allowed to analyze the cancer genome at very high sensitivity has subverted this notion and indicated that *CDKN1B* is mutated at a relatively high frequency in prostate cancer (PC) (20), small intestine neuroendocrine tumors (SI-NET) (21) and, especially, in luminal breast cancer (LBC) (22).

It is well established that, although all cancers carry many somatic mutations in their genomes, only a subset of those, known as driver mutations, confers clonal selective advantage to cancer cells and are implicated in oncogenesis. These mutations are opposed to the remaining ones that are referred to as passenger mutations (23). Mutations targeting *CDKN1B* have been considered driver in the above-mentioned sporadic tumors, as well as in the MEN familial syndromes.

In this review we aim to gather all reported mutations of *CDKN1B* in cancer and all related biological consequences, focusing our attention on human malignancies where pathogenic variants of *CDKN1B* are thought to give a selective clonal advantage and particularly discussing the significance of the mutations occurring in the IDR of the protein.

## *CDKN1B* mutational status in human malignancies

In order to have a comprehensive overview of the mutational status of p27 in all human malignancies, we interrogated the COSMIC, ICGC, cBioPortal, and TRANSFAC data portals and the current literature in PubMed, and then annotated all pathogenic mutations described for *CDKN1B* (Table 1, Supplementary Table 1). From our analysis we excluded the known polymorphism V109G that in some articles was reported as a mutation.

**Table 1:** CDKN1B mutations in hereditary syndromes.

**Tumor**	**Mutation**	**Location**	**Protein change**	**Reference**
MEN-like syndromes^*^	Frameshift	c.374_375delCT	p.S125^*^	(24–35)
	Frameshift	c.371delCT		
	Frameshift	c.59_77dup	p.K25fs	
	Missense	c.397C>A	p.P133T	
	Missense	c.25G>A	p.G9R	
	Missense	c.378G>C	p.E126D	
	Missense	c.678C>T	p.P69L	
	Missense	c.283C>T	p.P95S	
	Missense	c.357T>C	p.I119T	
	Nonsense	c.692G>A	p.W76^*^	
	Nonsense	c.595T>C	p.^*^199Qext^*^60	
	5′-UTR	c.-456_-453delCCTT		
	5′-UTR	c.-32_-29delGAGA		
	5′-UTR	c.-7G>C		
	5′-UTR	c.-29_-26delAGAG		
Familiar prostate cancer (FPC)	Missense	c.258G>C	p.E86D	(36–38)
	Missense	c.114C>T	p?	
	Missense	c.357T>C	p.I119T	
	5′-UTR	c.-79C>T		
	5′-UTR	c.-838C>A		
	3′-UTR	c.4149A>C		
	Promoter region	c.-1220A>C		
	Promoter region	c.-987C>T		

* *MEN-like syndromes include both in MEN1- and MEN4-syndrome*.

Among sporadic cancers, *CDKN1B* mutations were present in a significant proportion of analyzed PC, LBC, and SI-NET. We will therefore describe more in detail the mutations and their possible significance in these tumor types. Among neoplastic hereditary syndromes, we will focus our attention on MEN1 and MEN4, a MEN1-like syndrome, and on familial prostate cancer (FPC), since these are the two types of hereditary syndromes in which variants of *CDKN1B* have been reported to be more frequent and of higher pathological significance.

**Figure 2:**
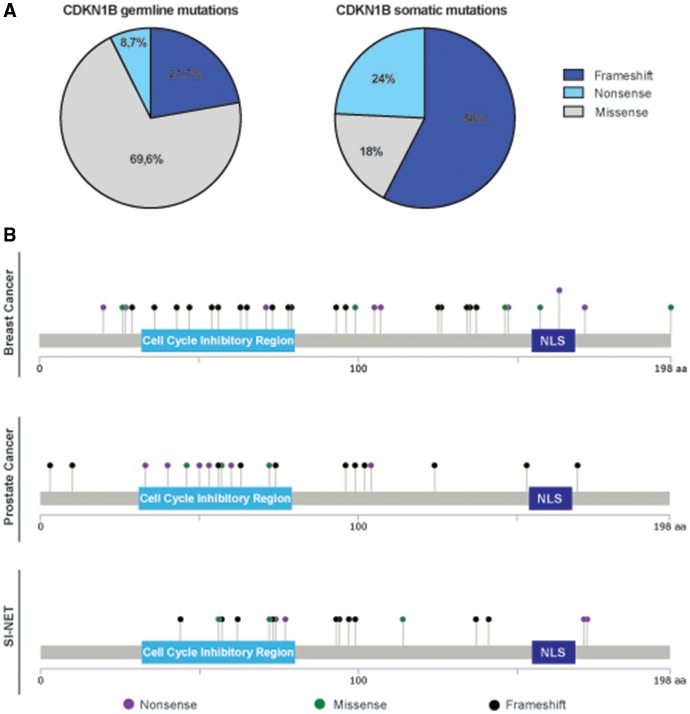
Distribution of germline and somatic *CDKN1B* mutations. **(A)** Pie graphs report the distribution of germline mutations in hereditary syndromes (left) and somatic ones in sporadic cancers (right). The percentage of each type of pathogenic variant (frameshift, including both insertion and deletions, missense, and nonsense) is reported. **(B)** Distribution of mutations identified in breast cancer (top), prostate cancer (middle), and SI-NET (bottom) are depicted along the sequence of p27 protein.

An intriguing observation was that we consistently noticed a different distribution and type of mutations in the *CDKN1B* gene, between hereditary syndromes and sporadic cancers. While in sporadic tumors somatic *CDKN1B* mutations are mostly located in the protein coding sequence (CDS), *CDKN1B* germline variants are fairly distributed along the whole sequence, both in the CDS and in untranslated regions (UTRs) (Table 1, Supplementary Table 1). Moreover, in hereditary syndromes most of germline mutations affecting the CDS are missense mutations, which result in a single amino acid change. Conversely, in sporadic cancers frameshift or nonsense mutations are largely predominant (Figure 2A). These mutations often result in the production of truncated forms of p27 protein lacking the C-terminus, which encodes for the p27 IDR.

## Hereditary syndromes

### Multiple endocrine neoplasia syndromes

Multiple endocrine neoplasia type 1 (MEN1) is a rare syndrome characterized by occurrence of parathyroid, pancreatic, and pituitary tumors. MEN1 syndrome can be either inherited, as an autosomal-dominant disorder, or developed during life as a sporadic event. Although MEN1 syndrome is generally due to mutations in the MEN1 gene (39), 5–10% of MEN1 patients display mutations in different genes (17, 40).

After the identification of a novel p27 mutation (p27 p.177fs) in a rat model of MEN syndrome, Pellegata and colleagues reported the discovery of a germline nonsense mutation (p.W76^*^) in heterozygosis in a patient suspected for MEN1 syndrome, but negative for MEN1 mutations (33, 41). This mutation led to a truncation of the resulting p27 protein that lost its nuclear localization signal (NLS) and was thereby retained in the cytoplasm where it could not fully exert its cell cycle inhibitory function. These studies, followed by the discovery of other MEN patients carrying *CDKN1B* mutation (see below), allowed the recognition of a novel human form of multiple endocrine neoplasia that includes MEN1-related tumors and that was named MEN4 (33).

Among the pathogenic variants reported in MEN4 families (Table 1), the vast majority are missense mutations located in the coding sequence. Some of described mutations have been functionally studied in *in vitro* models and are known to result in altered functionality of p27. For instance, it has been established that the nucleotide substitutions c.678 C>T (p.P69L) and c.283 C>T (p.P95S) disrupt p27 binding to GRB2 and CDK2, respectively, while the in frame deletions c.374_375delCT, the c.59_77dup, and 371delCT affect p27 nuclear localization (35). However, in some cases no clear alteration in p27 functions could be observed as a consequence of germline mutations identified in MEN4 families. This is, for example, the case of the c.397C>A (p.P133T) missense mutations that, using *in silico* analyses, have been predicted to be well tolerated in a physiological context (42). It is however intriguing to consider that both these mutations create new possible phosphorylation sites in the unstructured region, IDR of the protein, suggesting that an alteration of the binding to specific partners and/or of the engagement in non-canonical pathways could be occurring in the presence of these mutations. These are however only suggestive hypotheses that will need formal experimental demonstration to assess their validity.

Interestingly, there is also a group of germline *CDKN1B* mutations affecting p27 expression by altering the UTRs. For instance, the deletions c.-456_-453delCCTT and c.-32_29delGAGA lead to an impairment of the mRNA ribosome entry, on one hand lengthening the uORF (upstream Open Reading Frame), on the other disrupting the mRNA secondary structure (29, 31). Considering the notion that IDRs-containing proteins preferentially undergo on-site synthesis to ensures specific localization of the protein, we can well hypothesize that these mutations, altering uORFs or disrupting mRNA secondary structure, may alter p27 mRNA abundance, localization and asymmetric translation, eventually impacting on protein function and/or interaction.

Furthermore, it is interesting to note that also in the absence of p27 mutations, MEN1-3 syndromes might ultimately rely on altered p27 regulation and/or expression. In fact, MEN1 gene directly regulates *CDKN1B* transcription and TGFβ pathway (43–45), the latter eventually strongly impinging on p27 nuclear functions (46, 47). MEN2-3 syndromes are characterized by activating mutation of the RET proto-oncogene (33), that also controls p27 expression and functions (48, 49). Thus, together these data suggest that altered p27 functions, due to specific signaling alterations or to direct genetic inactivation, could be a common feature of MEN syndromes. Whether this role of *CDKN1B* in MEN syndromes is due to the peculiar functions exerted by p27 in these tissues or to the interaction between p27 and specific signaling transduction pathway(s) necessary for MEN onset, it is an issue that remains to be fully clarified.

### Familial prostate cancer

Several lines of evidence from clinical and experimental studies demonstrate that there is a linkage between the chromosomal location of the *CDKN1B* gene (12p13) and prostate cancer susceptibility in a considerable number of FPC (36). To test whether *CDKN1B* alteration contributes to an increased risk of prostate cancer, Chang and colleagues systematically sequenced 96 probands of families with prostate carcinoma. They identified a total of 10 germline *CDKN1B* variants, equally distributed in the ~800 bp promoter region, in the three exons and in the exon-intron junctions. Consulting the TRANSFAC database, the nucleotide changes c.-1220T>G, c.-987C>T, and c.-838C>A alter the binding sequences for transcription factors such as Pbx-1a, SP1, and E2F, respectively and could therefore affect the transcription of p27. The c.-79C>T was found to perturb one CpG site in a CpG island, known to be close to the transcription start site of a gene. The missense mutation p.E86D falls in the cell cycle inhibitory region and, as a consequence, could alter the binding of p27 protein with the cyclin/CDK complexes, although this hypothesis has not been experimentally confirmed. Among the other missense mutations described by the authors, they also propose that the p.I119T might alter the interaction between p27 and its negative regulator JAB1, which binds p27 between the amino acid residues 97 and 151 and is necessary for its cytoplasmic shuttling from the nucleus. Of note, they also identified the substitutions c.-79C>T and c.258G>C that fall in potentially exonic splice enhancer elements area^36^.

To sum up the results on germline *CDKN1B* mutations described so far, we can draw some interesting conclusions. First of all, homozygous germline loss of *CDKN1B* has never been found, suggesting that this possibility could be not compatible with life in humans. Second, germinal *CDKN1B* mutations principally determine an unbalance of the fine-tuned protein expression and/or localization, suggesting that small variation of p27 could be sufficient to promote transformation, at least in parathyroid, anterior pituitary adrenals, kidneys, and reproductive organs (MEN4 syndrome) or in prostate (FPC). This observation is substantially in line with the haploinsufficient role as tumor suppressor gene, ascribed to p27 many years ago, by the study of genetically modified mouse models.

### Somatic cancers

As mentioned above, prostate cancer (PC), luminal breast cancer (LBC), and small intestine neuroendocrine tumors (SI-NET) are the cancer subtypes in which *CDKN1B* mutations have been identified as driver genetic lesions in a significant percentage of cases (Supplementary Table 1). Common characteristic of these tumors is that they rely on hormones for their growth. Since these patients are typically treated with radio- and endocrine-therapies and can develop resistance to these first-line treatments and a role for p27 in the response to both radiation- and endocrine-therapy has been proposed, it will be important to ascertain whether *CDKN1B* mutational status in these tumors could act not only as genetic driver but also as predictor of therapy response.

### *CDKN1B* mutations in breast cancer

The downregulation of p27 protein was first described in breast cancer (BC), where it had significance of poor prognosis (50, 51). These data were then confirmed by several subsequent studies that, interestingly, identified high p27 expression not only as a marker of good prognosis but also as an independent predictor of responsiveness to hormonal therapy (52).

Even though the first attempts to identify *CDKN1B* mutation in BC were unsuccessful (53), more recent efforts by whole genome sequencing revealed that mutations affecting *CDKN1B* are present and can represent driver genetic lesions in the estrogen receptor (ER)-positive (LBC) subtype. The mutations identified in LBC are reported in Figure 2B. Ellis and colleagues sequenced 77 biopsies from LBC patients enrolled in two clinical trials testing aromatase inhibitors in neoadjuvant setting, to identify driver genetic lesions involved in drug response (22). Aromatase inhibitors are widely used to treat hormone-sensitive BC in the adjuvant- and in first line metastatic-setting (relapse of the disease after Tamoxifen treatment) (54), to prevent the biosynthesis of estrogens from androgens. The authors describe *CDKN1B* as one of the most significantly mutated gene in this cohort and, interestingly, several of the reported mutations resided in the C-terminal portion of p27 (p.E171^*^, p.K134fs^*^, p.P137fs^*^, p.Q163^*^; p.T198A) (22). In the same time, another group also sequenced the coding exons of p27 in 100 primary LBC tumors, reaching very similar conclusions and confirming two *CDKN1B* truncating mutations. Interestingly, for the first time this work reported the discovery of biallelic inactivation of *CDKN1B*, a feature very commonly found in recessive tumor suppressor genes (55). Some of the mutations described were at the N-terminus and were very likely affecting the cyclin/CDK binding domain of p27 (e.g., p.T42I) and/or determined a substantial loss of the protein functions (e.g., p.C29fs^*^12; p.E71^*^, and p.K73fs).

Among the different mutations described in LBC, there are two that have been extensively characterized in experimental models, namely the p.T198A and p.E171^*^ [(6, 8, 47, 56–59)]. The p.E171^*^ mutation results in the loss of 28 C-terminal amino acids, which are necessary for the interaction with stathmin. This interaction can impinge on mechanisms regulating cancer cell migration and also vesicular trafficking and receptor recycling [(6, 8, 57–59)]. The residue threonine 198 is important for p27 stability and to control cell motility and, accordingly, both these processes are altered in cells expressing the p.T198A mutant (47, 56, 59). It is interesting to note that both these mutations impinge on the ability of p27 to interact with the microtubule destabilizing protein stathmin (6, 59), suggesting that these interaction could have a role in tumor onset and/or progression. Intriguingly, both stathmin and p27 are IDPs and are fully regulated by post-transcriptional modifications. Their interaction seems to have a prominent role in the regulation of microtubule dynamics, eventually affecting several cellular functions such as proliferation, motility, and invasion.

Overall, these data indicate that, in primary LBC, *CDKN1B* mutations are mostly located in the C-terminal portion of p27, possibly contributing to alteration of protein localization and stability that, in turn, may profoundly affect cancer cell proliferation and motility.

Very recently, Yates and colleagues reported a deeper characterization of driver mutations by sequencing all coding exons of 365 cancer genes, in 756 primary tumors and in 227 samples from either distant or loco regional relapses. Then, they split the primary and relapse cohorts with respect to ER status. Only 15, out of the 365 genes analyzed, were identified as significantly mutated in primary tumors and/or recurrent/metastatic diseases. Among those, *CDKN1B* resulted significantly mutated in both ER-positive and -negative primary tumors, but not in metastatic/recurrent BC samples, overall confirming the pathological relevance of *CDKN1B* in BC and putting a particular accent on its involvement more during early steps of tumor initiation rather than during cancer progression (60).

### *CDKN1B* mutations in prostate cancer

PC is the most common malignancy in men worldwide. The standard therapy for PC is represented by radiotherapy coupled with surgical or medical castration. However, more than half of patients develop resistance to therapy and develop metastatic castration-resistant PC (CRPC) (61). Although several new anti-tumoral drugs are currently under clinical development, including compounds targeting the androgen receptor (AR) signaling axis, no therapy is currently curative for CRPC and no biological biomarker is currently available to predict patients' response (62). Notably, it has been demonstrated that p27 constrains PC growth in mice and the FOXA1 transcription factor, known to modulate AR-driven transcription, also induces the expression of *CDKN1B* (20). Recent genome wide studies provided an exhaustive overview of mutations occurring in local, metastatic or lethal PC [(20, 63, 64)]. In these reports, several genes whose products regulate AR function are frequently mutated or altered, both in primary and metastatic therapy-resistant tumors, suggesting that deregulation of the AR pathway could be an early event during prostate tumorigenesis (61). Barbieri *et al*. performed exome sequencing followed by paired-end, massive parallel sequencing of 112 primary prostate adenocarcinomas and matched normal samples, providing a cross section of PC genomes at diagnosis before treatment. *CDKN1B* has been identified as one of the most frequently somatically altered genes, mutated in three and deleted in sixteen tumor samples. The authors described one new missense mutation, p.E46G, which resides in the nuclear exportation signal of p27, and two new frameshift mutation, p.R169fs^*^ and p.R152fs^*^, both located in the nuclear localization signal of p27, leading to loss of the IDR region and likely protein displacement in the cytoplasm (63).

Robinson et al. conducted a systematic study of 150 metastatic pre-treated CRPCs to determine the landscape of somatic genomic alterations in this cohort. All alterations described in *CDKN1B* gene in this study, mainly frameshift mutations, were potentially driver and their overall incidence in the cohort reached the 4%. One frameshift variant was intronic, the others were the p.KGAC96fs and the p.P94fs, both likely leading to expression of a loss-of-function phenotype (65).

Grasso et al. reported the exome sequencing of 50 lethal-, heavily treated-, metastatic-CRPCs, obtained at autopsy of the patients. The authors describe a new frameshift mutation, the p.P101fs^*^, but this somatic variant did not result as driver in this cohort of clinical samples (66).

Beltran et al. investigated the mutational pattern of 45 formalin-fixed paraffin-embedded specimens from patients with localized PC, metastatic hormone-naïve PC and CRPC, but they did not find any considerable alteration in *CDKN1B* gene (62). However, given the heterogeneity and the small number of cases per cohort, it is conceivable that the presence of *CDKN1B* pathogenic variants could be underestimated in this study.

Altogether, mutations or deletions in *CDKN1B* have been often recognized as driver genetic lesions in primary and metastatic CPRCs, whereas their frequency in lethal metastatic CPRCs is not high enough to be considered clonal events. In support to this view, Baca *et al*. analyzed the clonal evolution of PC examining large genomic somatic rearrangements in 55 primary prostate adenocarcinoma and 2 metastatic Neuro Endocrine Prostate Cancer (NEPC). This study led to the conclusion that in PC large translocations and deletions arise in a highly interdependent manner. The authors name the emergence of these large genomic alterations as “chromoplexy” and suggest that chromoplexy disrupts multiple cancer genes in a coordinate manner, eventually leading to PC progression. Relevant to the topic of our review, Baca et al. reach the conclusion that *CDKN1B* inactivation by chromoplexy occurs frequently as a subclonal event and, therefore, could be linked with PC progression, possibly leading to genomic instability, proliferation and/or evasion of apoptosis (67).

Finally, the recently published atlas of metastatic tumors, collected from 10,336 patients (www.cbioportal.org/study?id=msk_impact_2017#) has reported the presence of *CDKN1B* mutations in 84 cases (somatic mutation rate = 0.7%). Altered allele frequency in these samples varied from 5 to 90%. Again, mutation of *CDKN1B* in metastatic tumors was restricted to some specific cancer types, including PC and BC. Among these, 5/500 (1%) PCs were found mutated and mutations included 1 missense, 1 nonsense, and 3 frameshift, at a frequency of 30–40%, overall confirming the driver role of *CDKN1B* mutation in, at least, a subset of PCs.

### *CDKN1B* mutations in small intestine-neuroendocrine tumors (SI-NET)

SI-NETs are rare tumors that derive from enterochromaffin cells of the neuroendocrine system of the gut. These cells contain a large amount of our body storage of serotonin. Clear information about how these tumors develop is limited (68), but recent studies tried to shed light on the genomic landscape of SI-NETs. Banck and colleagues have first analyzed 48 SI-NETs by massive parallel exome sequencing, both in tumors and in their normal tissue counterpart. They identified mutations in different genes implicated in cancer-related pathways, although none of them was consistently altered in a significant proportion of analyzed samples (69). However, in a subsequent study, Francis et al. profiled 55 tumors from 50 individuals, using a combination of whole-exome and whole-genome sequencing. The only gene identified as significantly mutated by this analysis was *CDKN1B*. In total, small insertion and deletions were found in the 10% of cases (Figure 2B). To confirm these data, they sequenced *CDKN1B* with a 800-fold mean coverage samples from two independent cohorts of SI-NETs, the one already sequenced by Banck et al. (48 samples) and another comprising 81 SI-NETs (21). Recurrent insertions and deletions leading to frameshift were identified in *CDKN1B*, in 14 out of 180 samples evaluated (8% of the individuals analyzed). In addition, hemizygous deletions, encompassing *CDKN1B* gene, were detected in 7 out of 50 patients. Altogether, the work from Francis *et al*. not only confirmed the previous findings but also found new heterozygous frameshift mutations in *CDKN1B*, strengthening the notion that this gene acts as a haploinsufficient tumor-suppressor in SI-NETs. These data were further confirmed in a larger study comprising 362 samples from 200 SI-NET patients. This work also led to the detection of *CDKN1B* mutations in 8% of the patients, making of *CDKN1B* the most frequently mutated gene in SI-NETs (70). However, it has to be highlighted that the expression of p27 protein did not correlate with *CDKN1B* mutational status and no clear difference in the clinical characteristics between *CDKN1B* mutated and *CDKN1B* wild type tumor carriers were found.

The works on SI-NETs give some interesting new insights on the possible role(s) and significance of *CDKN1B* mutations in human cancer. First, some *CDKN1B* mutations could be revealed only when NGS was carried out at high coverage (i.e., 800X) (21). If this was due to the subclonal nature of some *CDKN1B* mutations will be probably matter of future investigation. As for now, we can speculate that it may be worth using the same approach in LBC and PC, where total frequency reported for *CDKN1B* mutations is currently at 2%, but this percentage results from a whole-exome sequencing approach with relatively low coverage.

Second, the works on SI-NET confirm once again that *CDKN1B* acts as haploinsufficient tumor-suppressor, as already observed in different mouse models. Whether the same applies to other and more frequent cancers, such as LBC and PC, is something that certainly needs to be further investigated in the future.

## Conclusions

Our review of the literature and available datasets reporting the mutations of *CDKN1B* gene in human cancers indicates that these events are generally rare in sporadic cancer, with the exception of LBC, PC, and SI-NET. However, also in these tumors the frequency of *CDKN1B* mutation is below the 10% of the cases, partially supporting the old evidence that p27 expression is mainly deregulated at post-transcriptional level in human cancer.

A second relevant observation is that mutations encompassing *CDKN1B* gene are mainly subclonal. This has been proven in PC when large genomic rearrangements were studied (67).

The most intriguing observation is that ~80% of mutated tumor samples display nonsense or frameshift mutations at the C-terminus of the protein, leading to the formation of a truncated protein. This evidence has, in our opinion, two potential readings: the first is that the IDR of p27 plays relevant tumor suppressive activities in sporadic human cancer; the second is that the cytoplasmic displacement of p27, due to the loss of its nuclear localization signal (located in the C-terminus of the protein) is sufficient to drive tumor progression. Yet, this latter hypothesis is weakened by the notion that many functions of p27 have been ascribed to cytosolic interactions with other proteins, mediated by the C-terminal part of the protein. It is to note that, at least in PC and in LBC, *CDKN1B* mutations are less frequent in more aggressive/recurrent than in primary tumors, suggesting that the presence of these mutations could be linked to a more favorable scenario with respect to the one having a complete loss of function of p27 protein.

Furthermore, it is well known that p27 expression can be profoundly regulated by the expression of microRNAs, particularly miR-221 and miR-222. Therefore, it will be important to take into account that not only mutations occurring in 5′ UTR and in CDS, but also those in the 3′ UTR (for instance identified in one case of FPC) could contribute to alter the levels of p27 protein expression, eventually affecting cell transformation. Notably, while CDKN1B mRNA spans 2,535 base pair (bp), CDKN1B CDS only covers 596 bp, suggesting that regulatory regions present in the 5′ UTR (571 bp) and, especially, in the 3′ UTR (1,368 bp) could have a functional relevance. Moreover, we recently showed that p27 protein directly binds selected microRNAs eventually altering the proliferation of normal and cancer cells under specific culture condition. In particular, we identified an interaction between p27 and miR-223 that regulated cell proliferation/cell cycle arrest after achievement of cell-cell contact (71). Interestingly, we also verified that CDKN1B mutations identified in LBC could affect the ability of p27 to bind miR-223 (71).

Altogether, from the review of existing literature it is clear that many new insights, previously not hypothesized, on *CDKN1B* role and implications in cancer have been disclosed by the recent advent of next generation sequencing technologies. Nevertheless, many observations suggest that the interplay between CDKN1B mutations and p27 protein expression and function is far to be completely clarified and certainly merits further investigation, in particular with regard to the reciprocal modulation with non coding RNAs.

More experimental work will be necessary to fully clarify the role of p27 in tumor suppression and the significance of its genetic or functional deregulation in familial and sporadic human cancers, especially in those tumors driven by hormonal factors. Combining genetic and functional studies and using the most appropriate model systems will be mandatory to reach this important goal and establish at which extent each specific mutation identified does effectively impact on cancer initiation, progression, and/or on treatment response.

## Author contributions

MC, GM, MD, GB contributed to the revision of literature. MC, GM, MD, IS, BB, and GB wrote the paper. All authors read, corrected and approved the paper. MC, GM, MD equally contributed to this work.

### Conflict of interest statement

The authors declare that the research was conducted in the absence of any commercial or financial relationships that could be construed as a potential conflict of interest.
